# Stability of the Encoding Plasmids and Surface Expression of CS6 Differs in Enterotoxigenic *Escherichia coli* (ETEC) Encoding Different Heat-Stable (ST) Enterotoxins (STh and STp)

**DOI:** 10.1371/journal.pone.0152899

**Published:** 2016-04-07

**Authors:** Joshua Tobias, Astrid Von Mentzer, Patricia Loayza Frykberg, Martin Aslett, Andrew J. Page, Åsa Sjöling, Ann-Mari Svennerholm

**Affiliations:** 1 Department of Microbiology and Immunology, Institute of Biomedicine, University of Gothenburg, S-40530, Göteborg, Sweden; 2 Pathogen Genomics, Wellcome Trust Sanger Institute, Hinxton, Cambridge, CB10 1SA, United Kingdom; 3 Department of Microbiology, Tumor and Cell Biology, Karolinska Institutet, S-171 77, Stockholm, Sweden; University of Maryland School of Medicine, UNITED STATES

## Abstract

Enterotoxigenic *Escherichia coli* (ETEC), one of the most common reasons of diarrhea among infants and children in developing countries, causes disease by expression of either or both of the enterotoxins heat-labile (LT) and heat-stable (ST; divided into human-type [STh] and porcine-type [STp] variants), and colonization factors (CFs) among which CS6 is one of the most prevalent ETEC CFs. In this study we show that ETEC isolates expressing CS6+STh have higher copy numbers of the *cssABCD* operon encoding CS6 than those expressing CS6+STp. Long term cultivation of up to ten over-night passages of ETEC isolates harboring CS6+STh (n = 10) or CS6+STp (n = 15) showed instability of phenotypic expression of CS6 in a majority of the CS6+STp isolates, whereas most of the CS6+STh isolates retained CS6 expression. The observed instability was a correlated with loss of genes *cssA* and *cssD* as examined by PCR. Mobilization of the CS6 plasmid from an unstable CS6+STp isolate into a laboratory *E*. *coli* strain resulted in loss of the plasmid after a single over-night passage whereas the plasmid from an CS6+STh strain was retained in the laboratory strain during 10 passages. A sequence comparison between the CS6 plasmids from a stable and an unstable ETEC isolate revealed that genes necessary for plasmid stabilization, for example *pemI*, *pemK*, *stbA*, *stbB* and *parM*, were not present in the unstable ETEC isolate. Our results indicate that stable retention of CS6 may in part be affected by the stability of the plasmid on which both CS6 and STp or STh are located.

## Introduction

Enterotoxigenic *Escherichia coli* (ETEC) is a major cause of diarrhea among children in developing countries and in travelers to endemic areas [1]. Following ingestion of contaminated food or water, ETEC may colonize the small-bowel mucosa by means of surface structures, called colonization factors (CFs) or coli surface (CS) antigens [2].

ETEC diarrhea in humans results from secretion of a heat-labile toxin (LT) or a heat-stable toxin (STa), or both toxins [1]; STa is further sub divided into STh, which is only produced by human ETEC, or STp which can be produced by porcine as well as human ETEC isolates [3]. Among human ETEC, more than 25 CFs have been recognized, including CFA/I, CS1-8, CS12-15, CS17-21 as the most common CFs [1, 2]. The CFs are immunogenic proteins that bind to specific glycoprotein or glycolipid receptors on host epithelial cells [2, 4, 5], resulting in the adherence of ETEC to the host mucosa. Genes encoding the ETEC enterotoxins are plasmid-borne, and often linked to the CF genes [6, 7, 8].

The CF CS6 has been found to be expressed by clinical ETEC isolates with increased frequency in recent studies [1, 9, 10]. ETEC isolates typically harbor distinct combinations of toxin and CFs and CS6 positive isolates typically co-express LT+STh+CS5, LT+STp+CS4, LT+CS8 or only STp. Additional strains may also express STh and/or LT in combination with CS6. Several of these CS6 positive virulence variants are found in stable ETEC lineages with global distribution indicating high virulence potential and fitness [11]. Based on the high prevalence of CS6 expressing clinical isolates there has been considerable interest in using CS6 alone or in combination with other antigens in an ETEC vaccine [12, 13].

The genes associated with CS6 are expressed as a typical bacterial polycistronic operon that consists of four genes (c*ssA/B/C/D*) transcribed as a single mRNA, and located on a plasmid. *CssA* and *cssB* encode two heterologous structural subunits CssA and CssB. The CssC protein is a chaperone that assists in the folding of the two structural subunits; due to homology with other usher proteins, CssD has been ascribed an usher function responsible for transport of CssA and CssB to the cell surface [14, 15]. Most CFs are fimbrial or fibrillar; the structure of the CF CS6, is however not typical in being non-fimbrial, not protruding from the cell surface and most likely being expressed as an outer membrane protein [2]. Compared to other ETEC CFs, little is known regarding the regulation of CS6 expression. It has earlier been shown that deletion of CssC can significantly reduce levels of CssA, but not of CssB [15], and all the four gene products of CS6 operon are necessary for proper CS6 expression and cell adhesion [16].

In this study we compared several ETEC isolates expressing CS6+STp and CS6+STh, respectively with regard to stability of CS6 at both genotypic level (based on presence of genes *cssA* and *cssD*) and phenotypic level (surface expression) after multiple passages, and investigated whether stability of CS6 expression may be associated with presence of plasmid stabilizing genes.

## Materials and Methods

### Bacterial isolates and culture conditions

The ETEC isolates expressing CS6+STp or CS6+STh used in this study are listed in Table 1 and Table 2, respectively. Two additional strains, E58 expressing LT+STp+CS4+CS6 and E3003 expressing LT+STh+CS5+CS6, were also included in an initial experiment. Isolates were cultured in CFA broth [17], or LB medium supplemented with Kanamycin (Kan; 50 μg/ml), Chloramphenicol (Cm; 12.5 μg/ml), Tetracycline (Tet; 5 μg/ml) or Streptomycin (Strp; 50 μg/ml) when necessary. For long term cultivations of the isolates, 1–3 fresh colonies were inoculated into 25 ml of LB and incubated over-night at 37°C at 180 rpm. An inoculum of 25 microliters was transferred into 25 ml of fresh LB medium and incubated as above. The over-night culture was considered as the first passage, and additional passages were prepared similarly, as above, for up to 10 passages. An inoculum from each over-night culture was taken and used for phenotypic (surface) expression of CS6 by colony-blot [12], or genotypic expression of CS6, STp and STh by PCR [18].

**Table 1:** CS6+STp expressing ETEC isolates used in this study*.

Isolate (Lineage**)	Countryof origin	O group**	Virulence profile	CS6 Allele**	Accesion numbers**
E237 (L9)	Japan	27	STp	A6B3	ERS044477
E416 (L17)	Guatemala	27	STp	A6B3	ERS038936
E609 (NI)	Guatemala	169	STp	NA	
E830 (NI)	Guatemala	169	STp	NA	
E837 (NI)	Guatemala	27	STp	NA	
E927 (L9)	Egypt	159	STp	A7B3	ERS038952
E1245 (L7)	Egypt	169	STp	A6B3	ERS038963
E1329 (L7)	Kenya	169	STp***	A8B3	ERS206746
E1334 (L7)	China	NI	STp	A7B3	ERS055620
E1373 (L7)	Indonesia	169	STp	A7B3	ERS515514
E1392 (L17)	Indonesia	27	STp	A7B3	ERS038967
E1684 (L17)	Indonesia	27	STp	A6B3	ERS077748
E2695	Mozambique	27	STp	NA	
E3015 (L17)	Egypt	27	STp	A6B3	ERS077773
E5088 (L18)	Bangladesh	159	STp	A7B3	ERS055673

* All the examined ETEC strains in this study are from our own laboratory, i.e. the University of Gothenburg ETEC strain collection (and have been collected in collaborative studies.

** Derived from von Mentzer *et al* [11]

*** The isolate also expresses CS21 [11]

NA: Data not available (isolate was not sequenced)

NI: Not identified

**Table 2:** CS6+STh expressing ETEC isolates used in this study*.

Isolate (Lineage**)	Country of origin	O group**	Virulence profile	CS6 Allele**	Accesion numbers*
E114 (NI)	England	148	STh	NA	
E253	Japan	NI	STh	NA	
E996	Egypt	4	STh	A2B1	ERS077665
E1752 (L4)	Bangladesh	25	STh	A3B1	ERS077755
E1189 (L8)	Egypt	148	STh***	A1B1	ERS515513
E1355 (L8)	Egypt	148	STh***	A1B1	ERS077682
E1767	Bangladesh	NI	STh	NA	
E1784	Bangladesh	19	STh	A1B1	ERS038974
E2458	Kenya	NI	STh	NA	
E2545	Pakistan	25	STh	NA	

* All the examined ETEC strains in this study are from our own laboratory, i.e. the University of Gothenburg ETEC strain collection (and have been collected in collaborative studies.

** Derived from von Mentzer *et al* [11]

*** The isolate also expresses CS21 [11]

NA: Data not available (isolate was not sequenced)

NI: Not identified

### Colony blot assay for detection of surface (phenotypic) expression of CS6

A sample of 50–100 μl from each overnight culture (passage) was taken and spread on LB agar plates to get single colonies. One hundred single colonies were then examined for surface expression of CS6 by colony blot assay, as described [12] using specific MAbs against CS6 [19].

### DNA extraction

Isolates chosen for sequencing were grown on horse blood agar plates overnight at 37°C to detect potential contamination. Only pure ETEC cultures were used for DNA extraction. For Pacific Bioscience (PacBio) sequencing long intact strands of DNA must be obtained. The genomic DNA extraction was performed as follows: pure ETEC cultures were cultured in CFA broth overnight at 37°C followed by cell lysis using TE buffer (10 mM Tris and 1 mM EDTA pH 8.0) with 25% sucrose (Roche) and 100 mg/ml lysozyme (in 0.25 Tris pH 8.0) (Roche). Cell membranes were digested with Proteinase K and Sarkosyl NL-30 (Fisher) and RNase A was added to remove RNA molecules. To solubilize cell membranes 0.5M EDTA was added. A phenol-chlorofrom extraction was performed using a mixture of Phenol:Chrloroform:Isoamyl Alcohol (25:24:1) (Sigma) in phase lock tubes. To precipitate the DNA 99% ethanol was used followed by rehydration in genomic H_2_O. DNA concentrations of samples used for both real-time PCR and PacBio sequencing were measured using a NanoDrop spectrophotometer. An average of 484 ng/μl was used for PacBio sequencing.

### Real time PCR assays

Quantitative real time PCR was performed on an ABI 7500 (Applied Biosystems, Foster City, CA) using the double-stranded DNA-specific dye SYBR®Green I (Applied Biosystems, Warrington, UK) as detector, as described by the manufacturer. The primers and amplification conditions for the CS6 structural subunit gene *cssB* and the housekeeping gene *gapA* have previously been described [20]. Ct (cycle threshold) values were used to estimate the copy numbers of *cssB* located on the CS6 plasmids in comparison to the chromosomal gene *gapA*, and primers against these two genes had equal amplification efficiency; Illumina sequence analysis of the strains used both in this study and in a previous study [11] confirmed presence of one copy of *gapA* in the chromosome of the strains.

### PCR detection of CS6, STh and STp

PCR was applied for amplification of either *cssA* or *cssD* of CS6 and for STp or STh. All the PCR reactions were performed in 20 μl final volume containing 0.5 μl of the template DNA, 10 μl of ReadyMix (containing KAPA2G Fast HotStart DNA Polymerase, buffer, 0.2 mM of each dNTP, 1.5 mM of MgCl_2_, at the final concentration of 1.5 mM) (Techtum, Sweden). Template DNAs were prepared by centrifugation of over-night bacterial cultures at 16,000 *g* for 5 minutes. The pellets were resuspended in distilled water and boiled for 5 minutes and centrifuged at 16,000 *g* for 2 minutes. Following primers were used for PCR: *cssA* (5’-TCTAATTCT-TGCTTCATTCG, 5’-ACCAACCATAACCTGATC), *cssD* (5’-CAGAAATTCATGGAGTGGCTGA, 5’-CATGCTCCAGAAAATCCCAGA), *estA1* (STp) (5’-TCTTTCCCCTCTTTTAGTCAG, 5’-ACAGGCAGGATTACAACAAAG) and *estA2* (STh) (5’-TTCACCTTTCCCTCAGGATG, 5’-CTATTCATGCTTTCAGGACCA). The thermos-cycling conditions for all the PCRs were as follows: 95°C for 5 min, 95°C for 20 s, 55°C for 20 s, and 30 s at 72°C for 30 cycles, with a final 2 min extension at 72°C. Amplified samples were evaluated by 1.5% agarose gel electrophoresis in Tris-Acetate-EDTA buffer and EtBr staining.

### Mobilization of CS6 plasmid

PCR was applied to amplify an internal fragment from the CS6 operon, and cloned into the suicide plasmid pMT-Suicide-1 (supplied by M. Lebens) to construct the plasmid pJT-suicide-CS6-Cm. The constructed plasmid was replicated in *E*. *coli* S17-1 containing the Pir protein required for replication of the suicide vector, as well as the *tra* genes necessary for plasmid transfer. A conjugation between the *E*. *coli* S17-1 isolate, harboring the plasmid pJT-suicide-CS6-Cm, and the examined ETEC isolate was carried out on minimal medium plates containing Cm; on such plates, *E*. *coli* S17 cannot grow because it is auxotrophic to proline, and the ETEC isolate cannot grow due to its sensitivity to Cm. The conjugation resulted in an ETEC isolate with pJT-suicide-CS6-Cm integrated in the CS6 operon of the isolate. The clone was then conjugated with the *E*. *coli* isolate ED8654 harboring the helper plasmid pNJ5000 with Tet. One resulting clone, resistant to both Cm and Tet was selected and the presence of pJT-suicide-CS6-Cm in the CS6 operon of the clone was confirmed by PCR. The clone was then conjugated with *E*. *coli* TOP10, resistant to Strp, on a LB plate containing Cm and Strp. PCR was carried out to examine *E*.*coli* TOP10 clones harboring the CS6 plasmid, and one such clone was chosen and used for stability testing.

### DNA sequencing using the PacBio RSII

The DNA was stored in TE buffer and sequenced at Wellcome Trust Sanger Institute. Each isolate was sequenced using a single SMRTcell using the P6-C4 chemistry, to a target coverage of 40-60X using the PacBio RSII sequencer. The resulting raw sequencing data was manually *de novo* assembled using the PacBio SMRT analysis pipeline (https://github.com/PacificBiosciences/SMRT-Analysis) (version 2.3) utilizing the HGAP assembler [21]. The unfinished assemblies all produced a single, non-circular, chromosome plus some small contigs, some of which were plasmids or unresolved assembly variants. Using Circlator [22] (version 1.1.0), small self-contained contigs in the unfinished assembly were identified and removed, with the remaining contigs circularized. Quiver [21] was then used to correct errors in the circularized region by mapping corrected reads back to the circularized assembly. The final assembly was annotated using Prokka [23] (version 1.5). The annotation of plasmids pCss-E1189 and pCss-E1373 was confirmed by BLASTp.

## Results

### The copy number of CS6-containing plasmids in different ETEC isolates

Initially, different ETEC isolates expressing CS6 (alone or together with CS4 or CS5) and STa (i.e. either STh or STp), including E58 (LT+STh+CS4+CS6), E3015 (STp+CS6) and E3003 (LT+STh+CS5+CS6), were tested by RT-PCR to examine the copy number of the plasmid containing the genes for expression of CS6. This was carried out by comparing the copy number of *cssB* of the isolates and the housekeeping gene *gapA*, which has one chromosomal copy, by RT- PCR using DNA of the tested isolates. No marked difference in the Ct values for *gapA* was observed among the examined isolates, i.e. 12.46 (E58), 10.69 (E3015) and 8.86 (E3003), indicating that equal numbers of chromosomal copies were present in the samples. However, the isolates expressing STp, i.e. E58 and E3015, were shown to have considerably higher Ct values for *cssB*, 30.58 and 18.56, respectively, compared to the STh-expressing isolate E3003 (Ct 9.82), indicating that the STp-expressing isolates have lower copy numbers of the CS6-containing plasmid. This indicated that subsets of the individual bacteria that expressed STp had lost their plasmids.

### Stability of CS6 surface expression after repeated passages

To examine the stability of surface (i.e. phenotypic) expression of CS6 in strains producing STh or STp, all isolates listed in Table 1 and Table 2 were cultivated and passaged 10 times by over-night incubations. The surface presentation of CS6, examined by colony blot assay, was performed by examining at least 10 colonies from each isolate after the first and tenth passages. As shown in Table 3, the majority of the CS6+STp colonies expressed CS6 on their surface during the first passage. However, after 10 over-night passages the level of CS6 surface expression was considerably reduced; in most cases (9 out of 15) to ≥ 30% (Table 3).

**Table 3:** CS6 phenotypic expression of CS6+STp ETEC isolates.

	Stability of CS6 expression, as examined by colony blot analyses*	
Isolate (Lineage)	1^st^ passage	10^th^ passage
E237	52%**	0%
E416	100%	18%
E609	92%	3%
E830	82%	10%
E837	96%	60%
E927	100%	0%
E1245	100	30%
E1329	98%	32%
E1334	98%	94%
E1373	100	16%
E1392	100	54%
E1684	93%	0%
E2695	100%	96%
E3015	100%	0%
E5088	100%	98%

* Colony blot assay was performed as described in Materials and Methods.

** The values indicates the number of the colonies which expressed CS6, of the total number of examined colonies (n = 100).

Nearly all colonies (90%; 9 out of 10) of the CS6+STh isolates tested, expressed CS6 on their surface after the first passage and as many as 8 of 10 of the isolates had >94% CS6 positive colonies after 10 over-night passages (Table 4).

**Table 4:** CS6 phenotypic expression of CS6+STh ETEC isolates.

	Stability of CS6 expression, as examined by colony blot analyses*	
Isolate (Lineage)	1^st^ passage	10^th^ passage
E114	100%**	100%
E253	100%	100%
E996	96%	47%
E1752 (L4)	100%	94%
E1189 (L8)	100%	100%
E1355 (L8)	6%	0%
E1767	100%	100%
E1784	100%	100%
E2458	100%	100%
E2545	100%	100%

* Colony blot assay was performed as described in Materials and Methods.

** The values indicates the number of the colonies which expressed CS6, of the total number of examined colonies (n = 100).

No relation between ETEC linage background nor the allele type of CS6 and stability of CS6 surface expression was observed.

### Stability of CS6 surface expression, and association with ST genes in the isolates

To examine whether the instability of the phenotypic expression of CS6 was associated with loss of the CS6 genes and the co-expressed enterotoxins, PCR was applied on one isolate from each group, i.e. E927 (CS6+STp) and E1784 (CS6+STh). Ten colonies from each isolate were tested after the first and the tenth passage using primers against the structural subunit encoding gene *cssA* as well as the usher protein encoding gene *cssD* of CS6. All colonies of both isolates harbored the genes *cssA* and *cssD* after the first passage whereas only colonies of the CS6+STh isolate E1784 were positive for *cssA and cssD* after ten passages.

All the colonies were also examined by PCR for detection of genes encoding STp (*estA1*) and STh (*estA2*). This analysis showed that in colonies harboring *cssA* and *cssD*, the genes for STh or STp were also present. However, the *cssA* and *cssD* were found to be lost after ten overnight passages, which was associated with loss of the STp gene in colonies of strain E927. These results indicate that in the unstable isolate E927 loss of CS6 genes *cssA* and *cssD* is also associated with loss of the gene encoding STp, while the examined genes of CS6 and STh were still detected after 10 passages in the stable isolate E1784.

### Stability of CS6 surface expression after mobilization of the CS6-plasmid

It was recently reported that genes of the CS6 operon and of STh are located on the same plasmid in ETEC [8]. Hence, we examined whether the instability of CS6, both genotypic and phenotypic, in the CS6+STp isolates is affected by the host strain. This was done by mobilizing the CS6 plasmids from the unstable isolate E927 and from the stable isolate E1784, respectively, into an *E*. *coli* TOP10. While the TOP10(CS6+STh) isolate showed 100% surface expression of CS6, which correlated with presence of *cssA*, *cssD* and *estA2* after both one and ten passages, TOP10 (CS6+STp) was shown to lose the plasmid already during the first passage as examined by surface expression of CS6 and PCR for presence of *cssA*, *cssD* and *estA1* (data not shown).

### Comparison of sequences of an unstable and a stable CS6 plasmid

**Fig 1:**
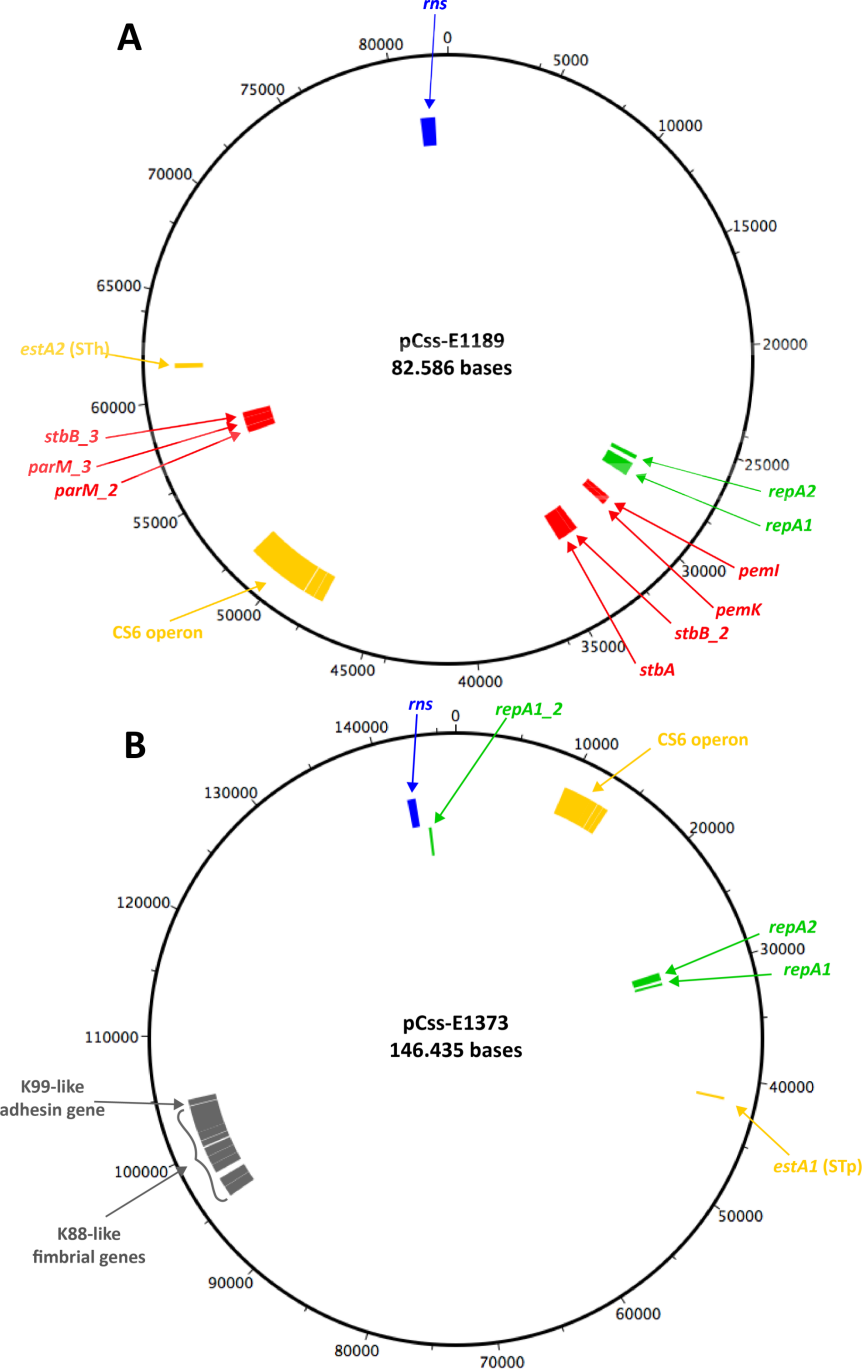
Graphical map of the plasmids pCss-E1189 (1A; Accession number LN908839), and pCss-E1373 (1B; Accession number LN908840). Virulence genes (in yellow), plasmid-specific genes for replication (in green) and for stability (in red), and the global regulator gene *rns* (in blue) are indicated. The plasmid-specific genes for stability found in pCss-E1189 were not found in plasmid pCss-E1373.

To examine whether the lack of genes, which encode products involved in plasmid stabilization, may have resulted in the instability of CS6 and STp expression, sequences of CS6-containing plasmids from the stable isolate E1189 (pCss-E1189, CS6+STh) and the unstable isolate E1373 (pCss-E1373, CS6+STp) were compared (Fig 1A and Fig 1B, respectively). In both plasmids, which were found to belong to FII compatibility group, the CS6 operon, as well as the genes encoding STh (E1189) and STp (E1373), were found (yellow). Also the *rns* gene, encoding a virulence regulator in ETEC [24], was found in both plasmids (blue). However, while plasmid-specific genes necessary for replication were found in both plasmids (green), only the pCss-E1189 (Fig 1A) was shown to harbor genes associated with plasmid stability, for example: *pemI*, *pemK*, *stbA*, *stbB* and *parM* (red). The plasmid pCss-E1373 (Fig 1B) did not harbor these genes. Notably, the size of pCss-E1373 was bigger than pCss-E1189, i.e. 146.435 bp vs. 82.586 bp, respectively, which partly is due to the presence of genes associated with animal ETEC CFs, K88 and K99 [25] in pCss-E1373. Identities of 38–99% and 51% between the genes of K88 and K99 found in pCss-E1373, respectively, were observed when compared with the protein sequences available at NCBI’s archival protein database.

## Discussion

In this study we compared the stability of CS6 surface expression and presence of the genes encoding the CssA and CssD, with genes *estA1* and *estA2* encoding STp or STh, respectively. As our initial experiment indicated that STh positive ETEC isolates may have higher copy numbers of CS6-encoding plasmids than those expressing STp, we selected several ETEC isolates that express CS6 together with STh or CS6+STp and compared or surface (phenotypic) expression of CS6. This comparison showed that CS6+STp ETEC isolates have considerably lower stability with respect to phenotypic expression of CS6, when examined after 10 over-night passages, than CS6+STh positive isolates. The genes necessary for assembly and expression for CS6 were initially shown to be located on a plasmid [26], and in a recent study CS6 and STh were shown to be located on the same plasmid [8]. We therefore investigated whether the loss of CS6 in the ETEC isolates examined in this study was associated with the loss of ST genes. We showed that while most strains that express CS6 and STh were stable after 10 passages, several of the CS6+STp strains had completely lost the examined genes encoding CS6 and STp after 10 cultivations.

An association between loss of surface expression of ETEC CFs, e.g. CFA/I or CFA/II (either CS1+CS3 or CS2+CS3) and loss of ETEC enterotoxins, either LT or STh, as examined by southern blot analyses has previously been reported [7]. That study showed that the loss of surface expression of the CFs was either due to loss of the plasmids containing these antigens, or by deletion of a segment of the plasmidial DNA encoding the toxins. In a later study [27] it was also reported that the loss of ST in the ETEC isolates examined was associated with deletions of DNA fragments in the plasmids. Our results, showing an association between loss of surface CS6 or its examined genes *cssA* and *cssD* with STp, which are located on the same plasmid, are in agreement with the results shown by the previous two studies. The CS6+STh and CS6+STp ETEC isolates examined in our study were from different geographical regions and also differed with regard to ETEC linage and CS6 allele types (Table 1 and Table 2). We did not observe that instability of CS6 expression was related to either lineage or the CS6 allele types, suggesting that other factors may play role in the instability of the CS6+STp plasmid.

In a recent study it was shown that CS6 with allele type A1B1 was associated with ETEC isolated from diarrheal in most cases, whereas the CS6 allele type A2B2 was predominantly found in asymptomatic controls [28]. In our study the CS6 allele type A1B1 was only observed amongst the stable CS6+STh ETEC isolates, and these isolates were isolated from patients with diarrhea.

All the ETEC isolates analyzed in the present study had expressed CS6 and ST when included in our ETEC strain collection. In addition, several ETEC lineages with stable strains expressing CS6+STp, which have caused diarrhea and spread globally, were recently reported [11]. E.g. in studies of ETEC disease in American travelers to Mexico and Guatemala CS6 STp ETEC were the most common isolates associated with diarrhea in 3 separate studies conducted during a period of more than 3 years [10]. This suggests that CS6+STp strains are indeed virulent but in-vitro culturing may trigger loss of CS6 and STp in unstable ETEC isolates.

To further investigate whether the nature of the plasmids may explain the different stabilities of CS6 in CS6+STh and CS6+STp ETEC strains, we mobilized the CS6 plasmids from a stable and an unstable ETEC isolate to the laboratory *E*. *coli* isolate TOP10. It was found that the mobilized plasmid from the CS6+STp isolate was lost already during the first over-night passage. However, the plasmid mobilized from a CS6+STh isolate was stable, i.e. *cssA* and *cssD* as well as the gene *estA2* (STh) were detected in this plasmid and the recombinant strain expressed CS6 on its surface after at least five passages. These results suggest that retention of the plasmid is mainly conferred by factors on the plasmid although we cannot exclude that the host strain may also affect the instability of CS6 plasmid.

We further investigated the content of the plasmids from a stable (CS6+STh) and an unstable (CS6+STp) isolate by PacBio sequencing. While the plasmid from the stable isolate harbored genes encoding plasmid stable inheritance as well as plasmid segregation proteins, e.g. *stbA*, *stbB* and *parM*, the plasmid from the unstable isolate did not harbor these genes. These results suggest that the observed instability of CS6+STp surface expression and loss of its *cssA* and *cssD* genes may be due to the lack of plasmid stabilizing proteins, resulting in the loss of the plasmids or at least fragments containing the examined genes of CS6 and STp. Among the genes detected in pCss-E1373, genes encoding K88 and K99 animal specific ETEC fimbriae [25] were found. We do not know, however, whether these genes are expressed or functional. Our finding of genes that are associated with the animal ETEC CFs K88 and K99 in the plasmid of the unstable ETEC CS6+STp positive isolate (E1373), and the fact that STp (porcine ST) may be of animal-origin, may suggest that plasmids from animal ETEC have been transferred to human ETEC. Furthermore, it is possible that the integration of different genes, i.e. K88 and K99 associated fimbrial genes and *rns* present in the unstable strain E1373 but not in the stable strain E1189, could affect the stability of pCss-E1373.

Altogether, our findings show that surface expression of CS6 and the examined genes encoding CssA and CssD are considerably less stable in CS6+STp than in CS6+STh positive ETEC strains. However, in spite of the frequent gene and plasmid loss we observed, STp CS6 strains are globally disseminated and associated with disease in humans indicating successful adaptation to the human host. These findings may have implications for the identification of CS6 and STp strains, with potential underestimation of STp+CS6 positive strains, which may be important in epidemiological studies.

## Abbreviations

CS: Coli Surface antigen
LT: Heat-labile enterotoxin
ST; STh, STp: Heat-stable enterotoxin
MAb: Monoclonal Antibody
PCR: Polymerase chain reaction
